# Predicting extreme thermal degradation of ascorbic acid (Vitamin C) using Bayesian-Inverse Weibull models: Applications in stability analysis and process optimization

**DOI:** 10.1371/journal.pone.0328554

**Published:** 2025-12-17

**Authors:** Rabia Azeem, Muhammad Aslam, Tahir Mehmood, Laila A. Al-Essa

**Affiliations:** 1 Department of Mathematics and Statistics, Riphah International University, Islamabad, Pakistan; 2 School of Natural Sciences, National University of Sciences and Technology, Sector H-12, Islamabad, Pakistan; 3 Department of Mathematical Sciences, College of Science, Princess Nourah bint Abdulrahman University, Riyadh 11671, Saudi Arabia; Sonarpur Mahavidyalaya, INDIA

## Abstract

Ascorbic acid (Vitamin C) is a thermally sensitive compound extensively used in pharmaceuticals, nutraceuticals, and food industries, where its degradation under high-temperature conditions can compromise product quality and efficacy. Accurate prediction of extreme thermal degradation events is crucial for ensuring stability, optimizing manufacturing processes, and meeting regulatory standards. However, traditional degradation models often fail to capture rare but critical degradation behaviors, resulting in inadequate risk assessments and suboptimal process controls.

In this study, we develop a Bayesian-Inverse Weibull modeling framework to predict extreme thermal degradation pathways of ascorbic acid under accelerated stress conditions. The Inverse Weibull distribution, known for its effectiveness in modeling heavy-tailed data, is integrated with a Bayesian hierarchical approach to incorporate prior knowledge, experimental data, and uncertainty quantification. This framework enables precise estimation of degradation thresholds, failure probabilities, and optimal storage and processing conditions.

Using experimental thermal degradation data, we validate the model and demonstrate its application in optimizing manufacturing processes to mitigate degradation risks. The results highlight the model’s superior capability in predicting rare degradation events, providing actionable insights for improving product stability, reducing waste, and ensuring regulatory compliance. This approach offers a robust tool for chemometric analysis and process optimization in industries reliant on thermally sensitive compounds like ascorbic acid.

## 1 Introduction

Ascorbic acid (Vitamin C) is a thermally sensitive compound widely utilized in pharmaceuticals, nutraceuticals, and the food industry due to its antioxidant properties and health benefits. However, its stability is highly susceptible to thermal degradation, posing significant challenges in maintaining product efficacy and shelf life. Under high-temperature conditions, ascorbic acid undergoes oxidative degradation, forming by-products such as dehydroascorbic acid and diketogulonic acid, which reduce its bioavailability and may introduce undesirable chemical reactions. Accurately predicting extreme thermal degradation events is crucial for optimizing storage conditions, improving manufacturing processes, and ensuring regulatory compliance.

Traditional degradation models, including the Arrhenius equation and first-order kinetic models, provide valuable insights into degradation dynamics but often fail to capture extreme degradation events accurately. These models primarily focus on average degradation rates, making them inadequate for risk assessment and process optimization, particularly in scenarios involving rare but critical degradation instances. To address this limitation, we propose a Bayesian-Inverse Weibull modeling framework to predict extreme thermal degradation of ascorbic acid under accelerated stress conditions. The Inverse Weibull distribution (IWD) is well-suited for modeling heavy-tailed data, effectively capturing the stochastic nature of degradation under extreme thermal stress. By integrating Bayesian inference, this framework incorporates prior knowledge, experimental observations, and uncertainty quantification, enabling precise estimation of degradation thresholds and failure probabilities.

A key component of our approach involves record values, particularly upper record values, which play a fundamental role in modeling extreme degradation events. Unlike traditional order statistics, record values provide a robust mechanism for analyzing extreme observations—an approach particularly relevant in chemometric analysis, food stability research, and pharmaceutical formulation studies. Chandler [1] first established the significance of record values in statistical inference, with subsequent advancements by Ahsanullah [2,3], Mubarik et al. [4], Yousaf et al. [5], and Alhamidah et al. [6] refining their applications. The use of upper record values enhances predictive modeling of extreme degradation scenarios, improving risk assessment and decision-making [7–9].

The Inverse Weibull distribution has been extensively applied in reliability engineering, medicine, and environmental sciences to model time-to-failure data, including wear-out periods and degradation processes. Calabria and Pulcini [10–14] developed Bayesian prediction methods for IWD, while Ahmadi et al. [15] explored its applications in generalized order statistics. More recent studies, such as those by Al-Duais et al. [16], have extended Bayesian inference to lower record values of the IWD, demonstrating its growing role in statistical modeling.

In this study, we develop and implement a Bayesian-Inverse Weibull modeling framework to predict extreme thermal degradation of ascorbic acid using upper record values. This approach enhances predictive accuracy and robustness in chemometric applications, offering practical utility in process optimization and stability evaluation. The findings contribute to a better understanding of degradation kinetics, with implications for improving storage conditions, minimizing thermal degradation losses, and supporting compliance with industry and regulatory standards.

## 2 Methodology

### 2.1 Thermal degradation data of ascorbic acid

This study employs a Bayesian-Inverse Weibull framework to predict extreme thermal degradation of ascorbic acid under accelerated stress conditions. Controlled experiments were conducted to capture degradation kinetics.

Experimental trials utilized high-purity ascorbic acid (≥99%), prepared in standardized aqueous solutions and stored in sealed glass vials to prevent oxidative degradation. Samples were subjected to thermal stress at $70^{\circ }C$, $85^{\circ }C$, and $100^{\circ }C$ in a programmable convection oven, with exposure times ranging from 10 to 120 minutes. Degradation was quantified using UV-Visible spectrophotometry at 265 nm and validated via high-performance liquid chromatography (HPLC) using a C18 column and acetonitrile-phosphate buffer (*pH* = 3.0) mobile phase. Data collection included triplicate measurements per condition, with preprocessing techniques applied to remove outliers and standardize the dataset.

### 2.2 Inverse Weibull distribution for thermal degradation modeling

The Inverse Weibull distribution is employed to model extreme thermal degradation behavior of ascorbic acid, accounting for heavy-tailed degradation characteristics. Parameter estimation is performed using both Maximum Likelihood Estimation (MLE) and a Bayesian approach.

#### 2.2.1 Maximum Likelihood Estimation (MLE).

MLE is utilized to estimate the parameters of the Inverse Weibull distribution by maximizing the likelihood function based on observed degradation data [17,18]. Suppose $X_{1},X_{2},\ldots ,X_{n}$ is a sequence of independent and identically distributed (iid) random variables from the Inverse Weibull distribution (IWD). Let *X*_*u*_ from $Y_{n}=X_{1},X_{2},\ldots ,X_{n}$ for n≥0 be declared as an upper record observation if its value is greater than that of all preceding values, where *X*_*u*(*n*)_ denotes the upper record value. Taking a sample size of *n* record observations $W_{n}=\left\{X_{u(1)},X_{u(2)},\ldots ,X_{u(n)}\right\}$, the likelihood function (LF) is given by:


$$L(\phi ,\gamma |x)=f\left(x_{u(n)};\phi ,\gamma \right)\prod_{i=1}^{n-1}\frac{f\left(x_{u(i)};\phi ,\gamma \right)}{1-F\left(x_{u(i)};\phi ,\gamma \right)}$$

$$=\frac{\phi^{n}\gamma^{n}\prod_{i=1}^{n}x_{u(i)}^{-(\gamma +1)}e^{-\phi \sum_{i=1}^{n}x_{u(i)}^{-\gamma }}}{\prod_{i=1}^{n-1}\left[1-e^{-\phi x_{u(i)}^{-\gamma }}\right]},$$ (1)

and the log-likelihood function is:


$$l(x;\phi ,\gamma )=n\log(\phi )+n\log(\gamma )-(\gamma +1)\sum_{i=1}^{n}\log x_{u(i)}-\phi \sum_{i=1}^{n}x_{u(i)}^{-\gamma }$$

$$-\sum_{i=1}^{n-1}\log\left(1-e^{-\phi x_{u(i)}^{-\gamma }}\right).$$ (2)

For simplification, the logarithm of the likelihood function is applied, and terms not containing the parameters are dropped. Differentiating (2) with respect to the parameters ϕ and γ and setting the resulting equations to zero yields:

$$\frac{\partial l(\phi ,\gamma |𝐱)}{\partial \phi }=\frac{n}{\phi }-\sum_{i=1}^{n}x_{u(i)}^{-\gamma }-\sum_{i=1}^{n-1}\frac{x_{u(i)}^{-\gamma }e^{-\phi x_{u(i)}^{-\gamma }}}{1-e^{-\phi x_{u(i)}^{-\gamma }}}=0,$$ (3)

$$\frac{\partial l(\phi ,\gamma |𝐱)}{\partial \gamma }=\frac{n}{\gamma }-\sum_{i=1}^{n}\log x_{u(i)}+\phi \sum_{i=1}^{n}x_{u(i)}^{-\gamma }\ln\left(x_{u(i)}\right)$$

$$-\sum_{i=1}^{n-1}\frac{\phi e^{-\phi }x_{u(i)}^{-\gamma }\ln\left(x_{u(i)}\right)}{1-e^{-\phi x_{u(i)}^{-\gamma }}}=0.$$ (4)

These equations are used to find the MLE estimates of the parameters ϕ and γ. Since (3) and (4) are not in closed form, an iterative numerical procedure, such as the Newton-Raphson method, is employed to solve them.

The MLE equations for ϕ and γ do not follow an exact distribution, making it challenging to derive exact confidence intervals. Therefore, a large-sample approximation is used. The asymptotic distribution of the MLE λ is given by $(\lambda -\hat{\lambda })\to N\left(\lambda ,I^{-1}(\lambda )\right)$, where $I^{-1}(\lambda )$ is the inverse of the observed Fisher Information matrix for the unknown parameters λ=(ϕ,γ):


$$I^{-1}={\left(\begin{array}{cc} -\frac{\partial^{2}l(\phi ,\gamma |x)}{\partial \phi^{2}} & -\frac{\partial^{2}l(\phi ,\gamma |x)}{\partial \phi \partial \gamma }\\ -\frac{\partial^{2}l(\phi ,\gamma |x)}{\partial \gamma \partial \phi } & -\frac{\partial^{2}l(\phi ,\gamma |x)}{\partial \gamma^{2}} \end{array}\right)|}_{\left(\phi ,\gamma )=(\hat{\phi },\hat{\gamma }\right)}^{-1},$$

which simplifies to:


$$I^{-1}=\left(\begin{array}{cc} \mathrm{var}(\hat{\phi }) & \mathrm{cov}(\hat{\phi },\hat{\gamma })\\ \mathrm{cov}(\hat{\gamma },\hat{\phi }) & \mathrm{var}(\hat{\gamma }) \end{array}\right).$$

The second-order partial derivatives are:


$$\frac{\partial^{2}l(\phi ,\gamma |𝐱)}{\partial \phi^{2}}=\frac{-n}{\phi^{2}}+\sum_{i=1}^{n-1}\frac{x_{u(i)}^{-2\gamma }e^{-\phi x_{u(i)}^{-\gamma }}}{{\left(1-e^{-\phi x_{u(i)}^{-\gamma }}\right)}^{2}}=0,$$

$$\frac{\partial^{2}l(\phi ,\gamma |𝐱)}{\partial \phi \partial \gamma }=\sum_{i=1}^{n}x_{u(i)}^{-\gamma }\ln x_{u(i)}-\sum_{i=1}^{n-1}\frac{x_{u(i)}^{-\gamma }e^{-\phi x_{u(i)}^{-\gamma }}\ln x_{u(i)}\left(\phi x_{u(i)}^{-\gamma }+e^{-\phi x_{u(i)}^{-\gamma }}-1\right)}{{\left(1-e^{-\phi x_{u(i)}^{-\gamma }}\right)}^{2}}=0,$$

$$\frac{\partial^{2}l(\phi ,\gamma |𝐱)}{\partial \gamma \partial \phi }=\sum_{i=1}^{n}x_{u(i)}^{-\gamma }\ln x_{u(i)}-\sum_{i=1}^{n-1}\frac{x_{u(i)}^{-\gamma }e^{-\phi x_{u(i)}^{-\gamma }}\ln x_{u(i)}\left(\phi x_{u(i)}^{-\gamma }+e^{-\phi x_{u(i)}^{-\gamma }}-1\right)}{{\left(1-e^{-\phi x_{u(i)}^{-\gamma }}\right)}^{2}}=0,$$ (5)

$$\frac{\partial^{2}l(\phi ,\gamma |𝐱)}{\partial \gamma^{2}}=-\sum_{i=1}^{n-1}\frac{\phi x_{u(i)}^{-\gamma }e^{-\phi x_{u(i)}^{-\gamma }}{\left(\ln x_{u(i)}\right)}^{2}\left(\phi x_{u(i)}^{-\gamma }+e^{-\phi x_{u(i)}^{-\gamma }}-1\right)}{{\left(1-e^{-\phi x_{u(i)}^{-\gamma }}\right)}^{2}}$$

$$-\frac{n}{\gamma^{2}}-\phi \sum_{i=1}^{n}x_{u(i)}^{-\gamma }{\left(\ln x_{u(i)}\right)}^{2}.$$

The 100(1−γ)% confidence limits for the parameters ϕ and γ are given by:


$$\left(\hat{\phi }-z_{\alpha /2}\sqrt{\mathrm{var}(\hat{\phi })},\hat{\phi }+z_{\alpha /2}\sqrt{\mathrm{var}(\hat{\phi })}\right),\;\left(\hat{\gamma }-z_{\alpha /2}\sqrt{\mathrm{var}(\hat{\gamma })},\hat{\gamma }+z_{\alpha /2}\sqrt{\mathrm{var}(\hat{\gamma })}\right),$$

where $z_{\alpha /2}$ is the critical value of the standard normal distribution. Due to the complexity of these equations, numerical methods are employed to solve them.

#### 2.2.2 Bayesian estimation.

A Bayesian framework is adopted to incorporate prior information and uncertainty quantification into the estimation process, enhancing predictive performance. Bayes’ theorem combines prior knowledge with current information to update beliefs, resulting in the posterior distribution. Gamma distributions are selected as independent priors for the shape and scale parameters, yielding the joint gamma prior distribution:

$$g(\phi ,\gamma )\propto \phi^{e-1}\exp(-f\gamma )\gamma^{g-1}\exp(-h\gamma ),\;\phi ,\gamma >0.$$ (6)

By combining (6) with the likelihood function (1), the posterior distribution is derived as:

$$p(\phi ,\gamma |𝐱)\propto \frac{\phi^{n+e-1}\gamma^{n+g-1}\prod_{i=1}^{n}x_{u(i)}^{-(\gamma +1)}e^{-\phi \left(f+\sum_{i=1}^{n}x_{u(i)}^{-\gamma }+h\gamma \right)}}{J_{0}\prod_{i=1}^{n-1}\left(1-e^{-\phi x_{u(i)}^{-\gamma }}\right)},$$ (7)

where the normalizing constant *J*_0_ is given by:

$$J_{0}=\int_{0}^{\infty }\int_{0}^{\infty }\frac{\phi^{n+e-1}\gamma^{n+g-1}\prod_{i=1}^{n}x_{u(i)}^{-(\gamma +1)}e^{-\phi \left(f+\sum_{i=1}^{n}x_{u(i)}^{-\gamma }+h\gamma \right)}}{\prod_{i=1}^{n-1}\left(1-e^{-\phi x_{u(i)}^{-\gamma }}\right)}\;d\phi \;d\gamma .$$ (8)

The marginal posterior distributions for ϕ and γ are obtained by integrating the joint posterior distribution:

$$p(\phi |𝐱)=\int_{0}^{\infty }\frac{\phi^{n+e-1}\gamma^{n+g-1}\prod_{i=1}^{n}x_{u(i)}^{-(\gamma +1)}e^{-\phi \left(f+\sum_{i=1}^{n}x_{u(i)}^{-\gamma }+h\gamma \right)}}{\prod_{i=1}^{n-1}\left(1-e^{-\phi x_{u(i)}^{-\gamma }}\right)}\;d\gamma ,$$ (9)

$$p(\gamma |𝐱)=\int_{0}^{\infty }\frac{\phi^{n+e-1}\gamma^{n+g-1}\prod_{i=1}^{n}x_{u(i)}^{-(\gamma +1)}e^{-\phi \left(f+\sum_{i=1}^{n}x_{u(i)}^{-\gamma }+h\gamma \right)}}{\prod_{i=1}^{n-1}\left(1-e^{-\phi x_{u(i)}^{-\gamma }}\right)}\;d\phi .$$ (10)

Bayesian estimators are obtained using the Metropolis-Hastings (MH) algorithm, a flexible Markov Chain Monte Carlo (MCMC) method introduced by Metropolis et al. [19] and extended by Hastings [20]. Since the Bayesian estimators (BEs) cannot be derived in closed form, a numerical procedure is required. The MH algorithm proceeds as follows:

1. Start with an initial value $\phi_{\tau }$. 2. Generate a candidate value $\phi^{\prime }$ from a proposal distribution $y(\phi_{\tau }/\phi^{\prime })$, where a gamma distribution is used as the transition kernel. 3. Compute the acceptance probability $w=\min(w_{1}w_{2},1)$, where:


$$w_{1}=\frac{y(\phi^{\prime }|𝐱)}{y(\phi_{\tau }|𝐱)},$$

$$w_{2}=\frac{y(\phi_{\tau }|\phi^{\prime })}{y(\phi^{\prime }|\phi_{\tau })}.$$

For symmetric distributions, *w*_2_ = 1. 4. Accept the candidate value with probability *w*:


$$\phi_{\tau +1}=\left\{ \begin{array}{cc} \phi^{\prime } & \text{with probability }w,\\ \phi_{\tau } & \text{with probability }1-w. \end{array}\right.$$

This process is repeated until the effect of initial values diminishes. To improve reliability, a burn-in period is applied by discarding initial samples. Convergence is monitored by ensuring the variance of the parameters decreases over iterations.

Posterior estimates are calculated as follows:

(i) Start with an initial value $\phi_{0}$.
(ii) Generate γ from the marginal distribution p(γ∣𝐱).
  (a) Compute the acceptance probability:
    $$y\left(\phi^{\left(i\right)},\phi^{\prime }\right)=\min\left(1,\frac{y\left(\phi^{\prime }|𝐱\right)y\left(\phi^{\left(i\right)}|\phi^{\prime }\right)}{y\left(\phi^{\left(i\right)}|𝐱\right)y\left(\phi^{\prime }|\phi^{\left(i\right)}\right)}\right).$$
  (b) Generate a random number *U* from a uniform distribution.
  (c) If $y\left(\phi^{\left(i\right)},\phi^{\prime }\right)\geq U$, set $\phi^{\left(j+1\right)}=\phi^{\prime }$; otherwise, retain $\phi^{\left(j\right)}$.
(iii) Collect $y\left(\phi_{j+1}|\phi_{j},\gamma_{j},\tau \right)$.
(iv) Repeat the process *N* times.
(v) Compute the Bayesian estimator of q(ϕ,γ) as:
  $$\frac{1}{K-B}\sum_{\tau =B+1}^{K}q\left(\phi_{\tau },\gamma_{\tau }\right),$$
  where *B* is the number of burn-in samples.

Posterior credible intervals are derived from the Bayesian model, providing a probabilistic measure of parameter uncertainty. Unlike classical confidence intervals, which rely on repeated sampling, Bayesian credible intervals offer a direct probabilistic interpretation. The marginal distribution of α is used to compute the Bayes limits $\left[\phi_{L},\phi_{U}\right]$ as follows [21]:


$$\int_{0}^{\phi_{L}}p_{1}(\psi |𝐱)\;d\psi =\frac{\delta }{2},\;\int_{\phi_{U}}^{\infty }p_{1}(\psi |𝐱)\;d\psi =\frac{\delta }{2}.$$

### 2.3 Record value prediction

Record values are analyzed to predict extreme degradation instances, aiding in the identification of critical stability thresholds.

#### 2.3.1 Classical prediction methods.

Traditional degradation prediction methods are implemented to compare their performance with the Bayesian-Inverse Weibull approach.

Consider the *n* upper record values $X_{u(1)},X_{u(2)},\ldots ,X_{u(n)}$ from the Inverse Weibull Distribution (IWD). The goal is to predict a future record value, denoted as *y* = *X*_*k*(*m*)_ where *m* > *n*. The joint predictive likelihood function of *y* = *X*_*k*(*m*)_ is given by Basak and Balakrishnan [22] as:


$$L(y,v,X)=\prod_{i=1}^{n}y\left(X_{u(i)},v\right)\frac{{\left[H(y;v)-H\left(X_{k(n)};v\right)\right]}^{m-n-1}}{\Gamma (m-n)}f(y;v),$$

where the cumulative hazard function *H*(*x*) is defined as:


$$H(x)=-\ln(S(x))=\ln\left[1-e^{-(\gamma /x{)}^{-\phi }}\right].$$

Based on the above likelihood function, the predictive likelihood for the IWD model is:


$$L(y;\phi ,\gamma )=\prod_{i=1}^{n}\frac{\phi }{\gamma }{\left(\frac{x_{u(i)}}{\gamma }\right)}^{-(\phi +1)}e^{-{\left(x_{u(i)}/\gamma \right)}^{-\phi }}$$

$$\times \frac{{\left[\ln\left(1-e^{-(\gamma /y{)}^{-\phi }}\right)-\ln\left(1-e^{-{\left(\gamma /x_{k(n)}\right)}^{-\phi }}\right)\right]}^{m-n-1}}{\Gamma (m-n)}$$

$$\times \left(\frac{\phi }{\gamma }\right)(y/\gamma {)}^{-(\phi +1)}e^{-(y/\gamma {)}^{-\phi }}.$$ (11)

The log-likelihood function is derived as:


$$l=(n+1)\log(\phi )-(n+1)\log(\gamma )+(\phi +1)\sum_{i=1}^{n}\log\left(\frac{x_{u(i)}}{\gamma }\right)$$

$$+\phi \sum_{i=1}^{n}\left(\frac{x_{u(i)}}{\gamma }\right)+(m-n-1)\log\left[\ln\left(1-e^{-{\left(\gamma /x_{k(n)}\right)}^{-\phi }}\right)\right]$$

$$-\ln\left(1-e^{-(\gamma /y{)}^{-\phi }}\right)-\log\Gamma (m-n)-(\phi +1)\log\left(\frac{y}{\gamma }\right)-{\left(\frac{y}{\gamma }\right)}^{-\phi }.$$

To estimate the parameters, the partial derivatives of the log-likelihood function with respect to ϕ, γ, and *y* are computed and set to zero:


$$\frac{\partial l}{\partial \phi }=\frac{n+1}{\phi }+\sum_{i=1}^{n}\log\left(\frac{x_{u(i)}}{\gamma }\right)+\sum_{i=1}^{n}\left(\frac{x_{u(i)}}{\gamma }\right)$$

$$+\frac{\left(m-n-1\right)}{\ln\left[\left(1-e^{-{\left(\gamma /x_{k(n)}\right)}^{-\phi }}\right)-\left(1-e^{-(\gamma /y{)}^{-\phi }}\right)\right]}$$

$$\times \left[\frac{e^{-{\left(\gamma /x_{k(n)}\right)}^{-\phi }}{\left(\gamma /x_{k(n)}\right)}^{-\phi }\ln\left(\gamma /x_{k(n)}\right)}{\left(1-e^{-{\left(\gamma /x_{k(n)}\right)}^{-\phi }}\right)}-\frac{e^{-(\gamma /y{)}^{-\phi }}(\gamma /y{)}^{-\phi }\ln(\gamma /y)}{\left(1-e^{-(\gamma /y{)}^{-\phi }}\right)}\right]$$

$$-\log\left(\frac{y}{\gamma }\right)-{\left(\frac{y}{\gamma }\right)}^{-\phi }\log\left(\frac{y}{\gamma }\right)=0,$$

$$\frac{\partial l}{\partial \gamma }=\frac{n+1}{\gamma }-(\phi +1)\sum_{i=1}^{n}\frac{\left(x_{u(i)}/\gamma^{2}\right)}{\left(x_{u(i)}/\gamma \right)}-\sum_{i=1}^{n}\left(\frac{x_{u(i)}}{\gamma^{2}}\right)$$

$$+\frac{\left(m-n-1\right)}{\ln\left[\left(1-e^{-{\left(\gamma /x_{k(n)}\right)}^{-\phi }}\right)-\left(1-e^{-(\gamma /y{)}^{-\phi }}\right)\right]}$$

$$\times \left[\frac{\phi e^{-{\left(\gamma /x_{k(n)}\right)}^{-\phi }}{\left(\gamma /x_{k(n)}\right)}^{-\phi -1}\left(1/x_{k(n)}\right)}{\left(1-e^{-{\left(\gamma /x_{k(n)}\right)}^{-\phi }}\right)}-\frac{\phi e^{-(\gamma /y{)}^{-\phi }}(\gamma /y{)}^{-\phi -1}(1/y)}{\left(1-e^{-(\gamma /y{)}^{-\phi }}\right)}\right]$$

$$+(\phi +1)\frac{\left(y/\gamma^{2}\right)}{\left(y/\gamma \right)}-\phi {\left(\frac{y}{\gamma }\right)}^{-(\phi +1)}=0,$$

$$\frac{\partial l}{\partial y}=\frac{\left(m-n-1\right)}{\ln\left[\left(1-e^{-{\left(\gamma /x_{k(n)}\right)}^{-\phi }}\right)-\left(1-e^{-(\gamma /y{)}^{-\phi }}\right)\right]}$$

$$\times \left[\frac{\phi e^{-(\gamma /y{)}^{-\phi }}(\gamma /y{)}^{-\phi -1}(1/y)}{\left(1-e^{-(\gamma /y{)}^{-\phi }}\right)}\right]$$

$$-(\phi +1)\frac{\left(1/\gamma \right)}{\left(y/\gamma \right)}+\phi \left(\frac{1}{\gamma }\right){\left(\frac{y}{\gamma }\right)}^{-(\phi +1)}=0.$$

An iterative numerical procedure is employed to solve these equations and obtain the maximum likelihood estimators (MLEs) for the predictors.

#### 2.3.2 Bayesian prediction methods.

Bayesian predictive distributions are utilized to forecast degradation behavior by integrating prior knowledge with experimental data. The influence of parameter variations on degradation predictions is examined to assess model robustness.

In the literature, numerous researchers have explored future record values within the Bayesian framework. For instance, Ahmadi et al. [15] and Madi et al. [23] have contributed significantly to this area. The objective is to estimate Bayesian estimators (BEs) and their credible intervals for the future record value *X*_*u*(*m*)_, for some *m*>*n*, with a specified confidence level. Ahsanullah [3] presented the conditional probability density function of *y* = *X*_*u*(*m*)_ given *z* = *X*_*u*(*n*)_ as follows:


$$g(y|z;\phi ,\gamma )=\frac{[\log H(z;\phi ,\gamma )-\log H(y;\phi ,\gamma ){]}^{m-n-1}}{\Gamma (m-n)}\times \frac{g(y;\phi ,\gamma )}{S(z;\phi ,\gamma )}$$

$$S(z;\phi ,\gamma )=1-e^{-(\gamma /z{)}^{\phi }}$$

$$S(y;\phi ,\gamma )=1-e^{-(\gamma /y{)}^{\phi }}$$

$$H(y)=-\ln(S(y))=\ln\left[1-e^{-(\gamma /y{)}^{-\phi }}\right]$$

$$g(y|z;\phi ,\gamma )=\frac{{\left[\log\left(1+e^{-(\gamma /z{)}^{\phi }}\right)-\log\left(1+e^{-(\gamma /y{)}^{\phi }}\right)\right]}^{m-n-1}}{\Gamma (m-n)}\times \frac{(\phi /\gamma )(\phi /\gamma {)}^{-(\phi +1)}e^{-(y/\gamma {)}^{-\phi }}}{\left(1+e^{-(\gamma /z{)}^{\phi }}\right)}$$

$$g(y|z;\phi ,\gamma )=\frac{(\phi /\gamma )(\phi /\gamma {)}^{-(\phi +1)}e^{-(y/\gamma {)}^{-\phi }}}{\Gamma (m-n)\left(1+e^{-(\gamma /z{)}^{\phi }}\right)}\times {\left[\log\left(1+e^{-(\gamma /z{)}^{\phi }}\right)-\log\left(1+e^{-(\gamma /y{)}^{\phi }}\right)\right]}^{m-n-1}$$

Generally, the Markov property is followed by the future record value *y* = *X*_*k*(*m*)_, given that $X=X_{u(1)},X_{u(2)},\ldots ,X_{u(n)}$, considering only the most recent record *Z* = *X*_*k*(*n*)_. The probability density function for the predictive model of *y* given *x* is given by:


$$g^{*}(y|x)=\int_{0}^{\infty }\int_{0}^{\infty }g(y|z,\phi ,\gamma )\times p(\phi ,\gamma |x)d\phi d\gamma$$

where g(y∣z,ϕ,γ) and p(ϕ,γ∣x) are defined above. Consequently, the 100(1−φ)% predictive intervals can be determined by solving:


$$\int_{y}^{y_{L}}g^{*}(y|x)dz=\frac{\varphi }{2},\;\int_{y_{U}}^{\infty }g^{*}(y|x)dz=\frac{\varphi }{2}$$

### 2.4 Sensitivity analysis

Sensitivity analysis examines how a model responds to variations in input parameters while keeping its structure unchanged. It identifies which inputs have the most significant impact on model predictions or results.

This analysis is valuable for decision-makers, as it highlights factors contributing most to uncertainty or variability in the model’s outcomes. For instance, in some cases, a small input change leads to a significant shift in the model’s output, while in others, input variations have negligible effects. A model exhibiting the latter behavior is considered less sensitive to changes in prior parameters.

### 2.5 Simulation study

This section analyzes and compares the Maximum Likelihood Estimators (MLEs) and Bayesian Estimators (BEs) of the Inverse Weibull Distribution (IWD) for upper record values using different sample sizes. It also details the method for generating upper record samples from the IWD.

Abdi and Asgharzadeh [24] addressed record value generation from any continuous distribution. Their procedure for generating $U_{1},U_{2},\ldots ,U_{n}$ upper record values from a continuous distribution is given by:


$$U_{i}={\left(\frac{-1}{\phi }\log\left({\;V}_{i}\right)\right)}^{-1/\gamma }$$

Pakhteev and Stepanov [25] proposed another method for finding upper record values. According to their approach, first, draw the i.i.d. random variables $R_{1},R_{2},\ldots ,R_{n}$ from the continuous distribution *G*(.). Assuming that $R_{1},R_{2},\ldots ,R_{n}$ follow the Markov property, the probability function is given by:


$$P\left(Z(n+1)\leq z_{n+1}|Z_{\left(n\right)}=z_{n}\right)=\frac{G\left(z_{n+1}\right)-G\left(z_{n}\right)}{1-G\left(z_{n}\right)}\;\left(z_{n+1}>z_{n}\right)$$

For our distribution, this formula converts to:


$$Z_{i}=\frac{-1}{\phi }\log{\left(\prod_{i=1}^{n}U_{i}\left(e^{{\left(-\gamma /x_{\left(n\right)}^{u}\right)}^{\phi }}\right)+e^{{\left(-\gamma /x_{\left(n\right)}^{u}\right)}^{\phi }}\right)}^{-1/\gamma }$$

Since *U*_*i*_ follows a uniform distribution and *Z*_*i*_ represents record values generated from the IWD, both methods yield nearly identical record values, demonstrating their accuracy.

## 3 Results

This section presents the simulation results, along with the estimation and prediction analyses related to the thermal degradation of ascorbic acid (Vitamin C).

Table 1 reports the maximum likelihood estimates (MLEs) of the parameter *ϕ* along with their standard errors (SE) and confidence intervals for different sample sizes (n=10,20,30,40,50). The simulations were performed by setting ϕ=1 and γ=1. The results indicate that as the sample size increases, the MLEs stabilize, and the standard errors decrease.

**Table 1:** MLEs for ϕ.

n	ϕ		γ	
	Mean	SE	Mean	SE
10	0.9606	0.0043	0.9679	0.0171
20	0.9607	0.0042	0.9681	0.0169
30	0.9607	0.0041	0.9683	0.0168
40	0.9609	0.0041	0.9687	0.0168
50	0.9609	0.0040	0.9691	0.0166

**Fig 1:**
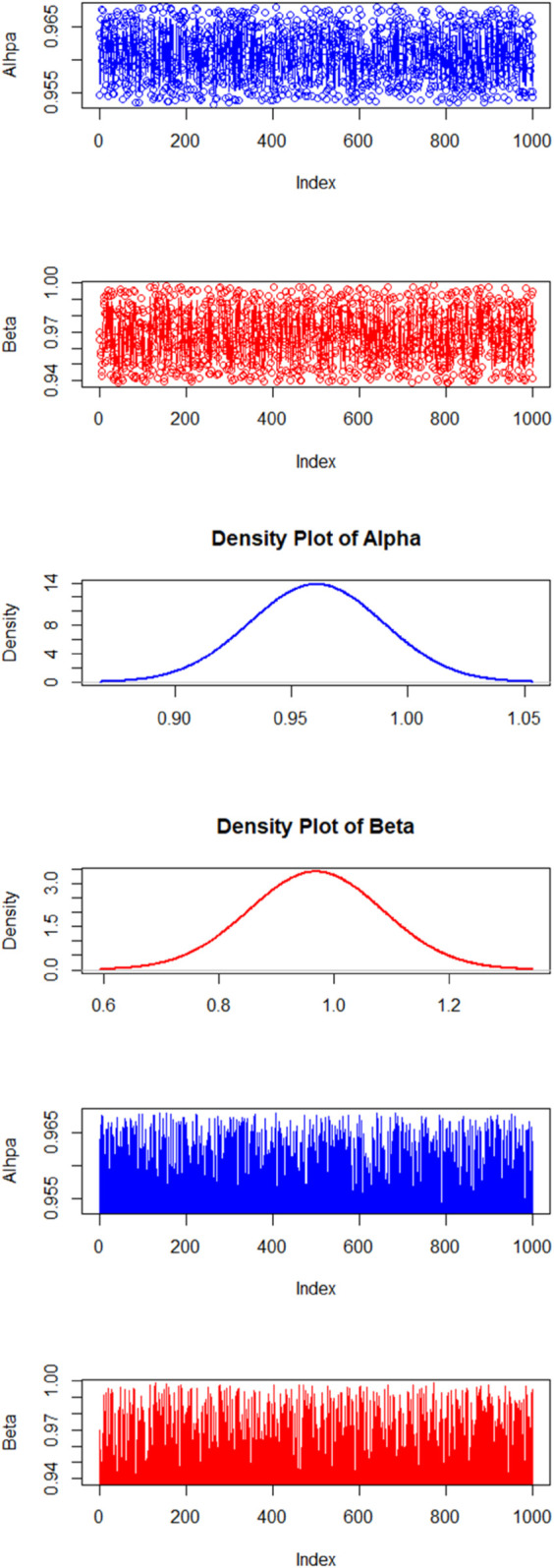
Efficiency checks of the MCMC method through density, history, and trace plots for both parameters. The graphical patterns indicate that the Metropolis-Hastings (MH) algorithm performs effectively. However, the density plot of *γ* exhibits a slight skewness.

For *n* = 10, Fig 1 shows the efficiency of the Metropolis-Hastings (MH) algorithm through density, history, and trace plots for both parameters. The trace plots confirm that the Markov Chain Monte Carlo (MCMC) samples exhibit good mixing. The history plots suggest that the chain has reached equilibrium, while the density plots reveal the posterior distribution of the parameters. A slight skewness is observed in the density plot of *γ*.

Table 2 presents the Bayesian estimates (BEs) for *ϕ* using informative priors. The table includes standard errors, Monte Carlo (MC) errors, and confidence intervals. The results show that as the sample size increases, the estimates become more precise, with reduced standard errors and narrower confidence intervals. Similar trends are observed for the parameter ***γ*, as detailed.** in Table 3.

**Table 2:** Estimates for ϕ using informative priors.

n	Estimate	SE	MC Error	2.5 %	Median	97.5 %
10	0.7152	0.0012	0.00123	0.7135	0.575	0.8445
20	0.7540	0.0008	0.00083	0.7513	0.5832	0.9114
30	0.7900	0.0007	0.00058	0.7886	0.5986	1.0026
40	0.9047	0.0006	0.00046	0.8991	0.6029	1.2004
50	0.9570	0.0005	0.00039	0.9449	0.6404	1.4194

**Table 3:** Bayesian estimates for γ using informative priors.

n	Estimate	SE	MC Error	2.5 %	Median	97.5 %
10	0.4490	0.0018	0.00031	0.4358	0.2797	0.6907
20	0.5720	0.0017	0.00029	0.5649	0.4099	0.7892
30	0.7361	0.0014	0.00028	0.7283	0.5607	0.9595
40	0.8614	0.0011	0.00026	0.8551	0.6492	1.0883
50	0.9911	0.0009	0.00025	0.9873	0.7681	1.2416

The findings indicate that Bayesian estimation using informative priors provides accurate parameter estimates with increasing precision as sample sizes grow. The results confirm the robustness of the proposed methodology for estimating parameters related to thermal degradation processes.

**Fig 2:**
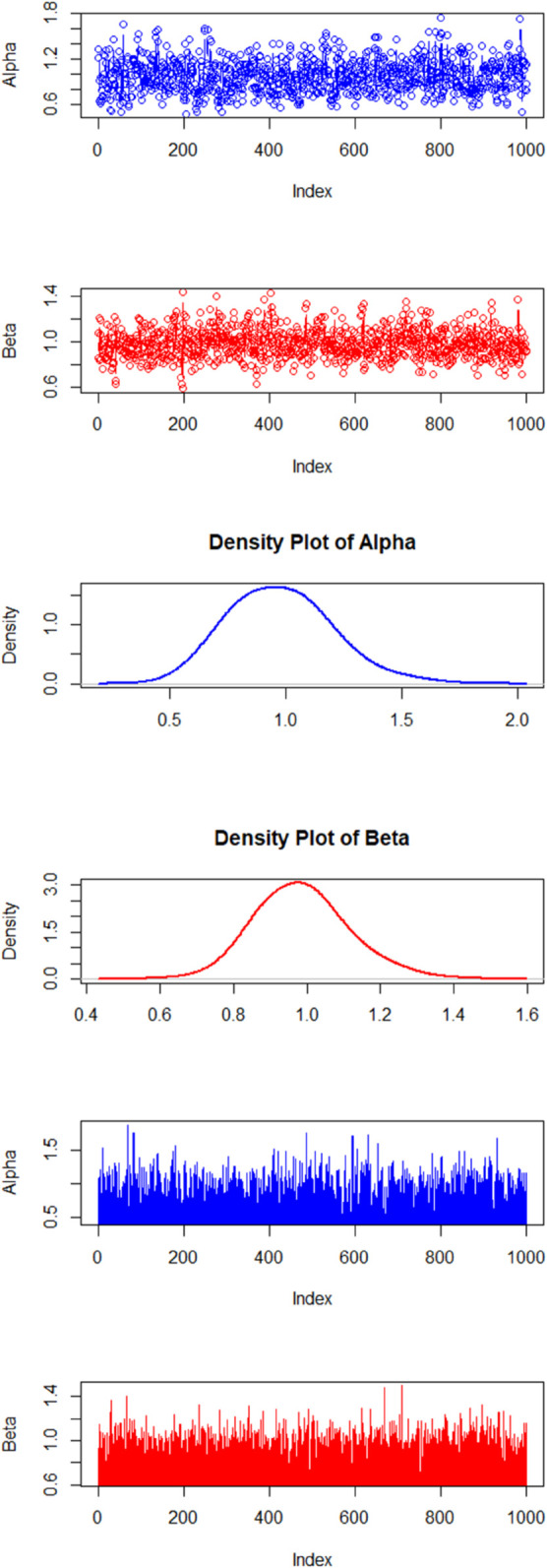
The figure shows the density, history, and trace plots for the posterior distributions. The density plots reveal a symmetric distribution, indicating that the parameter estimates are well-behaved. The visual inspection of the trace and history plots further supports the convergence of the MCMC chains, demonstrating that the suggested method is working effectively.

By fixing the hyperparameters as e=f=3 and g=h=3, results mean  = 1 and variance  = 0.333. Tables 4 and 5 show the results for the estimates of *ϕ* and *γ*, respectively. There are little fluctuations in the estimates but the standard error decreases with increase in the sample size. Columns 3, 4, and 5 show the median, 2.5 and 97.5 percentiles. The proposed method converges quite well, shown by the trace and history plots with n=50, in Fig 2. It shows the density, history, and trace plots for the posterior distributions. The density plots reveal a symmetric distribution, indicating that the parameter estimates are well-behaved. The visual inspection of the trace and history plots further supports the convergence of the MCMC chains, demonstrating that the suggested method is working effectively.

**Table 4:** Bayesian estimates of ϕ using Non-Informative Prior (NIP).

n	Estimate	SE	MC Error	2.5 %	Median	97.5 %
10	0.7567	0.0021	0.00154	0.7559	0.6727	0.8408
20	0.7880	0.0017	0.00083	0.7887	0.6775	0.8913
30	0.8056	0.0016	0.00060	0.8048	0.6896	0.9312
40	0.8957	0.0014	0.00048	0.8957	0.7013	1.0752
50	1.0060	0.0010	0.00042	0.9993	0.7319	1.3588

**Table 5:** Bayesian estimates of γ using Non-Informative Prior (NIP).

n	Estimate	SE	MC Error	2.5 %	Median	97.5 %
10	0.5457	0.0017	0.00032	0.5392	0.4301	0.7072
20	0.6662	0.0015	0.00028	0.6615	0.5381	0.8283
30	0.7971	0.0014	0.00028	0.7912	0.6487	0.9845
40	0.8909	0.0013	0.00024	0.8856	0.7434	1.0668
50	0.9903	0.0012	0.00023	0.9876	0.8216	1.1900

The selection of non-informative priors e=f=1 and g=h=0.6 leads to a posterior distribution with a mean of 1.66 and a variance of 2.778. The estimates and their associated standard errors are summarized in Tables 4 and 5. In these tables, the second column presents the Bayes estimates, which appear to be consistent across the different sample sizes. Additionally, the standard errors exhibit a decreasing trend as the sample size increases for both parameters *ϕ* and *γ*, which reflects improved precision in the estimates with larger sample sizes.

Sensitivity analysis plays a crucial role in Bayesian statistics, as it helps to assess how the choice of prior distribution influences the posterior estimates. In this study, we conducted a sensitivity analysis by varying the values of the hyperparameters and examining their effect on the posterior distributions of the parameters *ϕ* and *γ*. Table 6 presents the results of this analysis.

**Table 6:** Sensitivity analysis at different hyperparmeters.

n	Mean	SE	MC Error	2.50 %	Median	97.5 %
ϕ(2,2)	0.9870	0.1036	0.0018	0.9855	0.7906	1.1975
γ(2,2)	0.9934	0.1132	0.0017	0.9879	0.7867	1.2294
ϕ(1.5,1.5)	0.9876	0.1016	0.0016	0.9856	0.7884	1.1882
γ(1.5,1.5)	0.9922	0.1100	0.0014	0.9846	0.7964	1.2285
ϕ(1.2,1.2)	0.9889	0.1040	0.0017	0.9869	0.7923	1.2008
γ(1.2,1.2)	0.9917	0.1139	0.0015	0.9839	0.7867	1.2365
ϕ(1,1)	0.9922	0.1048	0.0012	0.9905	0.7914	1.2020
γ(1,1)	0.9890	0.1146	0.0014	0.9831	0.7813	1.2375
ϕ(1,0.75)	0.9938	0.1057	0.0013	0.9924	0.7907	1.2037
γ(1,0.75)	0.9901	0.1135	0.0015	0.9836	0.7824	1.2322
ϕ(2,1)	0.9997	0.1040	0.0010	0.9982	0.8000	1.2056
γ(2,1)	0.9989	0.1135	0.0011	0.9939	0.7964	1.2374

The first column shows different combinations of prior hyperparameters, where each row corresponds to a different setting of the prior variance. For instance, the first row represents the posterior estimates when the prior variance is 0.5. The subsequent rows show estimates for prior variances between 0.5 and 1.0, while the last two rows correspond to prior variances greater than 1.0.

It is observed that as the prior variance increases, the standard errors also increase, reflecting greater uncertainty in the parameter estimates. This trend is especially noticeable in the last two rows of the table, where the prior variance is more than 1. In contrast, for the non-informative prior, the sensitivity to changes in the prior variance is relatively lower.

In addition, a random sample of 50 future record values was generated by assuming ϕ=1 and γ=1. Using these values and assuming the hyperparameters *e* = *f* = 3 and *g* = *h* = 3, the predicted 51st record value is 1.6136. The conditional median prediction for this value is 1.6139, and the 95% credible interval for the future record value is (1.60997, 1.61724).

This segment presents the application of record values on the thermal degradation study of ascorbic acid.

Samples were subjected to thermal stress at three different temperature levels: $70^{\circ }C$, $85^{\circ }C$, and $100^{\circ }C$, within a programmable convection oven. Exposure times ranged from 10 to 120 minutes for each condition.

**Fig 3:**
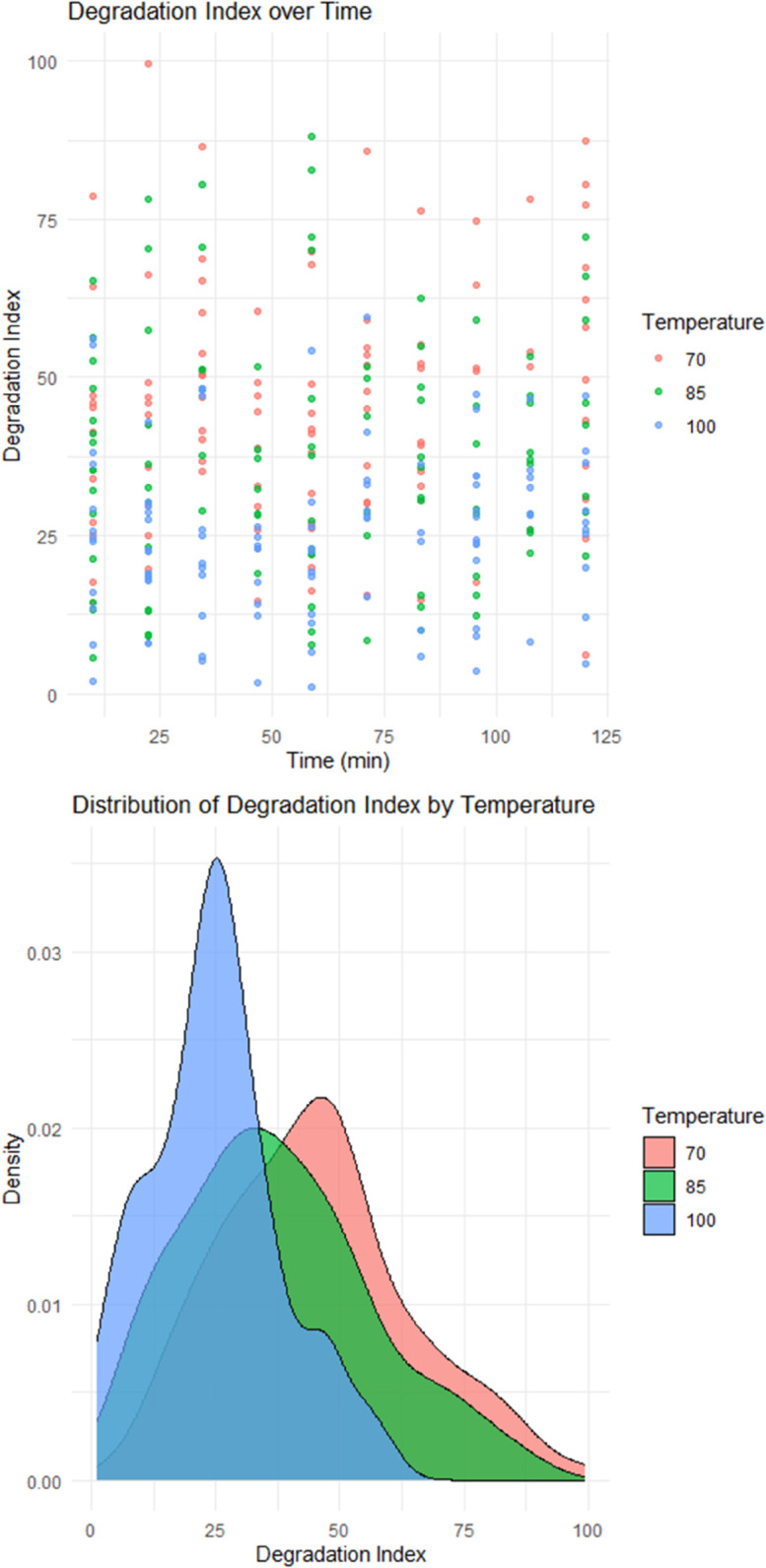
The distribution of degradation index by temperature and time.

For each temperature level, 100 samples were tested. The distribution of degradation rate is presented in Fig 3. The aim was to develop a reliable model for predicting the degradation rate of ascorbic acid based on varying temperature and exposure conditions. The parameter estimation based on the thermal data is presented in Table 7.

**Table 7:** BEs of ϕ and γ based on thermal degradation data.

	MLE	BEIP	BENP
**Estimate**			
*ϕ*	9.4135	2.4029	2.5358
*γ*	7.7320	0.0130	0.0130
**SE**			
*ϕ*	1.4662	0.0089	0.0091
*γ*	4.0841	0.0004	0.0006

The Degradation Index reflects the concentration of intermediate colored products formed during ascorbic acid breakdown. At 70 ^°^C, the reaction proceeds at a moderate rate, allowing these intermediates to accumulate and produce a higher absorbance peak. In contrast, at 85 ^°^C the degradation proceeds more rapidly through both primary and secondary pathways, converting intermediates into colorless end-products before they can build up, which leads to a lower observed peak. This behavior aligns with thermal degradation kinetics observed in previous studies, where higher temperatures promote faster degradation, reducing the visibility of intermediates.

**Fig 4:**
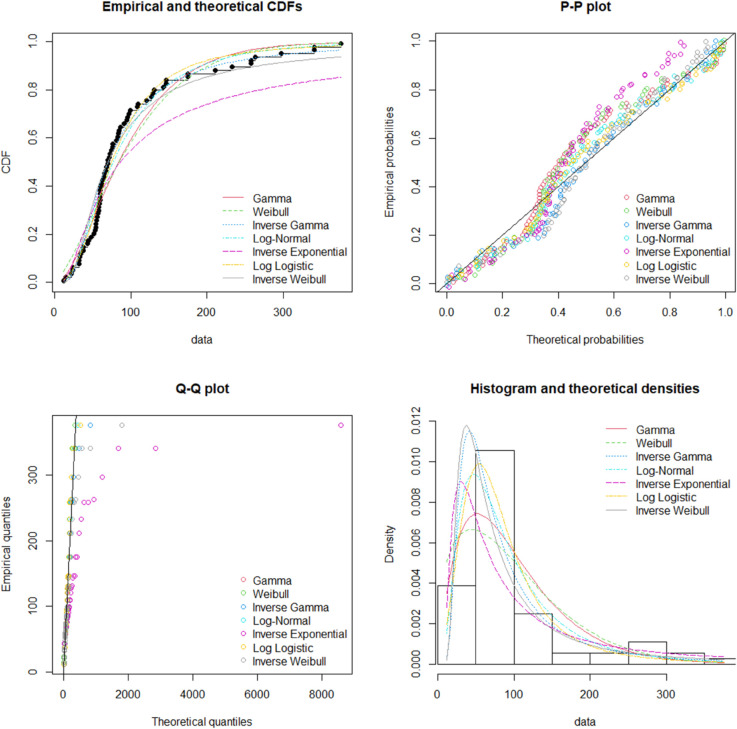
Visual assessment of goodness of fit for thermal degradation data.

Five different tests (Anderson Darling (AD), Kolmogorov-Smirnov (KS), Bayesian Information Criterion (BIC), Cramér-von Mises (CVM), and Akaike Information Criterion (AIC)) were employed to fit the data. The Inverse Weibull Distribution (IWD) was compared to other distributions, including Gamma, Inverse Gamma, Weibull, Inverse Exponential, and Log Normal. The IWD demonstrated the best fit to the data, meeting the criteria of all tests. The presentation of goodness of fit is presented in Fig 4 and Table 8.

**Table 8:** Data fitting results for thermal degradation data of ascorbic acid.

Distribution	AIC	BIC	KS- Statistic	AD- Statistic	CVM- Statistic
Gamma	792.49	797.05	0.1383	1.8937	0.3546
Weibull	798.29	802.84	0.1463	2.3715	0.4307
Inverse- Weibull	789.22	793.77	0.1341	0.9307	0.1589
Log- Normal 784.67	789.22	0.0955	0.8403	0.1539	
Inverse- Exponential	807.34	809.62	0.1844	4.5867	0.8307
Log- Logistic	783.92	788.48	0.0865	0.6411	0.1031

Using the real data set, the Bayesian intervals for the parameters of *ϕ* and *γ* are estimated as (1.86520,2.9622) and (0.0110,0.0162), respectively. Based on the calculated values, the next 101st future record is predicted to be 95.2 if the temperature is 70 C, 93.7 if the temperature is 85 C, and 101.4 if the temperature is 100 C. Furthermore, the 95% predictive interval for the future record value are (89.4,99.21), (90.4,100.01) and (97.21,105.49). The future record value and the conditional median prediction both fall within the 95% predictive interval, supporting the accuracy of our predictions.

## 4 Conclusion

This study successfully employed a Bayesian-Inverse Weibull framework to model the thermal degradation of ascorbic acid under accelerated stress conditions. By subjecting ascorbic acid samples to various temperatures and exposure times, we were able to collect comprehensive experimental data that informed the degradation kinetics. The results demonstrated that the Inverse Weibull distribution provided the best fit to the degradation data when compared with other common distributions such as Gamma, Weibull, and Log Normal, as evidenced by multiple goodness-of-fit tests including AIC, BIC, KS, AD, and CVM.

The Bayesian estimation technique was applied to derive credible intervals for the key parameters of the degradation model, with the results providing a reliable prediction for the degradation rate of ascorbic acid under varying thermal stress conditions. The future record prediction and its corresponding 95% predictive interval offered a solid basis for anticipating the degradation behavior of ascorbic acid, confirming the robustness and precision of the developed model.

These findings contribute valuable insights into the degradation processes of ascorbic acid, with potential applications in food science, pharmaceuticals, and other industries where ascorbic acid stability is of significant concern. While the Bayesian-Inverse Weibull approach offers strong predictive power and flexibility with record data, it also demands careful prior selection and incurs higher computational costs. These insights have been incorporated to provide readers with a balanced and critical understanding of the model’s practical utility.Future studies may aim to broaden the scope of analysis by integrating other environmental stressors, such as light and oxygen exposure, and by extending the application of the Bayesian-Inverse Weibull model to a wider range of thermally sensitive compounds.
